# Influence of radiation treatment technique on outcome and toxicity in anal cancer

**DOI:** 10.1007/s13566-017-0326-3

**Published:** 2017-08-22

**Authors:** Elisha T. Fredman, May Abdel-Wahab, Aryavarta M.S. Kumar

**Affiliations:** 1 Department of Radiation Oncology, Seidman Cancer Center, University Hospitals Cleveland Medical Center, 11100 Euclid Ave, Cleveland, OH 44106 USA; 2 Department of Nuclear Sciences and Applications, International Atomic Energy Agency’s (IAEA) Division of Human Health, Vienna, Austria; 3 University Hospitals Parma Seidman Cancer Center, Parma, OH USA

**Keywords:** Anal cancer, Radiation, IMRT, 3D, Toxicity

## Abstract

**Objective:**

Intensity-modulated radiation therapy (IMRT) has largely supplanted three-dimensional conformal radiation (3D-CRT) for definitive anal cancer treatment due to decreased toxicity and potentially improved outcomes. Convincing data demonstrating its advantages, however, remain limited. We compared outcomes and toxicity with concurrent chemotherapy and IMRT vs 3D-CRT for anal cancer.

**Methods:**

We performed a single-institution retrospective review of patients treated with IMRT or 3D-CRT as part of definitive mitomycin-C/5-fluorouricil-based chemoradiation for anal cancer from January 2003 to December 2012.

**Results:**

One hundred sixty-five patients were included, with 61 and 104 receiving IMRT and 3D-CRT, respectively. Overall, 92.7% had squamous cell carcinoma. The mean initial pelvic dose was 48.3 and 44 Gy for IMRT and 3D-CRT, respectively. Complete response, partial response, and disease progression rates were similar (IMRT 83.6, 8.2, 8.2%; 3D-CRT 85.6, 6.7, 7.7%; *p* = 0.608, *p* = 0.728, *p* = 0.729). There was no significant difference in overall survival (*p* = 0.971), event-free survival (*p* = 0.900), or local or distant recurrence rates (*p* = 0.118, *p* = 0.373). IMRT caused significantly less acute grade 1–2 incontinence (*p* = 0.035), grade 3–4 pain (*p* = 0.033), and fatigue (*p* = 0.030). IMRT patients had significantly fewer chronic post-treatment toxicities (*p* = 0.008), outperforming 3D-CRT in six of eight toxicities reviewed. Though total treatment length was comparable (43.6 and 44.5 days), IMRT recipients had fewer (27.9 vs 41.3% of patients, *p* = 0.89), shorter treatment breaks (mean 2.9 vs 4.1 days, *p* = 0.229).

**Conclusion:**

This report represents the largest series directly comparing concurrent chemotherapy with IMRT vs 3D-CRT for definitive treatment of anal cancer. IMRT significantly reduced acute and post-treatment toxicities and allowed for safe and effective pelvic dose escalation.

## Introduction

Eight thousand eighty people in the USA are expected to be diagnosed with anal cancer in 2016, resulting in 1080 deaths [1]. Despite comprising only 1.5% of all gastrointestinal tumors, the rates of diagnosis have been increasing in the USA and globally [2, 3]. The current standard of care is definitive chemoradiation, with abdominoperineal resection (APR) reserved for salvage therapy [4, 5].

Advancements in radiation treatment over time have attempted to better optimize its delivery to potentially reduce toxicity and allow for maximal dose escalation. Historically, two-dimensional techniques based on surface anatomy and bony landmarks were used to deliver radiation for anal cancer. Until recently, three-dimensional conformal radiation (3D-CRT) was the most commonly utilized treatment modality, incorporating CT imaging data to better identify the intended target. Intensity-modulated radiation therapy (IMRT) followed, which can be used to design still more conformal radiation fields. By modulating the intensity of each beam delivered, a dose can be designed to target the concavities and convexities of a tumor volume, thereby further reducing dose to adjacent tissues.

Within the last few years, IMRT has been shown to be more efficacious and less toxic than traditional 3D-CRT for multiple disease sites [6–9]. Smaller case series have reported similar advantages of IMRT in the context of anal cancer, but conclusive data remain limited [10–15]. Though these studies present survival and toxicity outcomes, even fewer are comparative in nature [12, 13]. Furthermore, a wide range of results have been reported thus far, from a significant survival benefit with IMRT to more modest results of decreased toxicities and fewer treatment breaks [11, 12, 15, 16]. Since IMRT is both substantially more expensive and technically demanding, it is critical to rigorously evaluate the scope of its benefit. We therefore present a comparison of patients treated for anal squamous cell carcinoma with 3D-CRT vs IMRT, with an emphasis on acute toxicity, chronic post-treatment sequelae, and clinical outcomes.

## Methods

Internal Review Board approval was obtained to perform a retrospective review of all anal cancer patients evaluated at our institution between January 2003 and December 2012. Patients had biopsy-confirmed invasive anal cancer treated with definitive chemoradiation using either 3D-CRT or IMRT, including patients with regional nodal involvement. Patients were excluded if they had distant metastases or if treatment intent was palliative. Of the 249 patients initially identified, 165 were eligible. All patients underwent complete staging using contrast-enhanced CT, MRI, or FDG-PET.

Pertinent demographic information and tumor characteristics were collected based on previously identified risk factors for the development of anal cancer [17]. Tumor factors included histological type, grade, AJCC stage (7th edition), and distance from the anal verge measured on imaging and exam. Acute toxicities during treatment were collected from weekly on-treatment and radiation completion notes, while post treatment, late toxicity were collected from radiation and medical oncology follow-up documentation.

### Chemotherapy

Patients received 2 cycles of concurrent infusional 5-fluorouracil (5-FU)-based cytotoxic chemotherapy. In the majority of instances, treatment entailed combination 5-FU and bolus mitomycin-C (MMC) per the Nigro protocol [18]. Based on practice variability among medical oncologists, 10.9% of patients received 5-FU alone, 5-FU/cisplatin, oral Xeloda, 5-FU/cisplatin/vinblastine, or an etoposide-based treatment.

### Radiation therapy

All patients underwent CT simulation, either prone with a belly board or supine. Non-IMRT radiotherapy planning followed classic two-dimensional radiotherapy borders for mini-pelvic fields, cone downs, and tumor boosts [19]. Electrons were used to supplement dose to the inguinal lymph nodes. IMRT contours and dose constraints were according to RTOG 05-29 [20]. Briefly, contours including the gross tumor, and elective lymph node regions (inguinal, internal iliac, external iliac) were defined using imaging studies with appropriate margin expansions for CTV and PTV. Total dose was 50.4 Gy for T2, 54 Gy for T3–T4, and 45 Gy for elective lymph node regions.

### Toxicity

Acute toxicity was defined as treatment effect occurring between radiation therapy start and 8 weeks after radiation treatment completion [20]. Late-developing effects were defined as occurring from 8 weeks through 1 year post treatment, the longest period of follow-up for the majority of IMRT recipients. Acute toxicities assessed were localized pain, diarrhea, fatigue, dermatitis, hematologic changes, incontinence, enterocolitis, colitis, proctitis, fistula formation, and anal/vaginal stenosis. Late toxicities included intractable diarrhea, anal/vaginal stenosis, enterocolitis, proctitis, anal ulcers, fistula formation, wound dehiscence, and refractory pain. The Common Terminology Criteria for Adverse Events v3.0 (CTCAEv3) quantified toxicity [21].

### Treatment response and follow-up

Tumor response was assessed by physical exam and follow-up imaging. Recurrence was determined clinically by physical exam. Biopsy was only performed if the lesion was clinically indeterminate. A complete response (CR) was recorded if there was no evidence of residual tumor, partial response (PR) if there was a response of at least 30% on exam or imaging compared with presentation, or progressive disease (PD), defined as an increase in tumor size on exam or imaging. Follow-up was every 3–4 months for the first year, and every 4–6 months thereafter without evidence of clinical recurrence.

## Results

### Patient factors

Of 165 patients, 61 (37%) received IMRT and 104 (63%) received 3D-CRT. Since the start of 2010, 76.7% of patients received IMRT, rising to 83.3% after 2012. Both treatment groups were demographically comparable (Table 1), and the majority had a favorable ECOG performance status of 0–1 (85.2 and 94.3%, respectively). Though HIV infection was more common in the IMRT group (13.1 vs 5.8% in 3D-CRT), there was a comparable percentage of patients in each group receiving chronic immunosuppressive therapy (14.8% in the IMRT group and 10.6% in the 3D-CRT group).

**Table 1:** Demographic factors (*n* = 165)

	Value (%)	
	IMRT (*n* = 61)	3D-CRT (*n* = 104)
Sex		
Male	21 (34.4)	37 (35.6)
Female	40 (65.6)	67 (64.4)
Age		
Mean	58.8 ± 10.9	55.9 ± 11.5
Range	38–89	26–85
Performance status		
ECOG 0–1	52 (85.2)	98 (94.3)
2	7 (11.5)	2 (1.9)
3	2 (3.3)	4 (3.8)
KPS 100 − 90	38 (62.3)	68 (65.4)
80–70	21 (34.4)	32 (30.8)
≤ 60	2 (3.3)	4 (3.8)
BMI		
< 18.5	0 (0)	3 (2.9)
18.5–24.9	28 (45.9)	40 (38.5)
25–29.9	22 (36.1)	26 (25)
≥ 30	11 (18)	35 (33.7)
Smoking		
Yes	36 (59.0)	69 (66.3)
Pack years (average)	26.1 ± 21.5	32.7 ± 22.0
HPV positive	10 (16.4)	11 (10.6)
HIV positive	8 (13.1)	6 (5.8)
Immunosuppressive therapy	9 (14.8)	11 (10.6)
Tumor type		
Squamous cell	56 (91.8)	97 (93.3)
Adenocarcinoma	3 (4.9)	4 (3.8)
Other	2 (3.3)	3 (2.9)
Anal verge distance		
Mean	0.44 ± 0.99 cm	0.61 ± 1.22 cm
Range	0–4 cm	0–6 cm
Spanning verge	25 (41.0)	49 (47.1)
T stage		
1	8 (13.1)	12 (11.5)
2	34 (55.7)	53 (51.0)
3	13 (21.3)	27 (26.0)
4	6 (9.8)	12 (11.5)
N stage		
0	23 (37.7)	59 (56.7)
1	14 (23.0)	19 (18.3)
2	13 (21.3)	16 (15.4)
3	11 (18.0)	10 (9.6)
M stage		
0	54 (88.5)	96 (92.3)
1	7 (11.5)	8 (7.7)
Tumor grade		
1	9 (14.8)	15 (14.4)
2	39 (63.9)	64 (61.5)
3	13 (21.3)	25 (24.0)

*IMRT* intensity-modulated radiation therapy, *3D-CRT* 3-dimentional conventional radiation therapy, *ECOG* Eastern Cooperative Oncology Group, *KPS* Karnofsky performance status

### Tumor factors

Squamous cell carcinoma was the most common histology in both IMRT and 3D-CRT groups (91.8%, 93.3%). Adenocarcinoma, cloacogenic, small cell, mucinous, and adenosquamous tumors were rarer histologies. Seventy-seven percent of the tumors in both groups were T2–3 (Table 1).

### Chemotherapy

One hundred fifty-two of 165 (92.1%) patients received 2 cycles of concurrent infusional 5-FU-based chemotherapy. Of these 152 patients, 135 (88.8%) were treated with combination 5-FU and bolus MMC according to the Nigro protocol, 46 (75.4%) in the IMRT group, and 89 (85.6%) in the 3D-CRT group.

### Radiation treatment

In the IMRT group, 55.9% of patients were treated supine and 44.1% prone, while 84.8 and 15.2% of 3D-CRT patients were treated supine and prone, respectively. Patients in the IMRT group received a higher mean pelvic dose compared to 3D-CRT (48.3 vs 44 Gy) with a mean tumor boost of 9.6 and 12.7 Gy, respectively. The mean total dose, number of fractions, and overall length of treatment were the same for both groups. 3D-CRT patients exhibited a wider range of treatment length compared to IMRT (25–110 vs 31–71 days) (Table 2).

**Table 2:** Radiation treatment regimen

	Mean (SD)		Range	
	IMRT	3D-CRT	IMRT	3D-CRT
Pelvic dose	48.3 (6.2)	44 (6.6)	59.4–30.6	63–30
Boost	9.6 (4.3)	12.7 (4.7)	23.4–3.6	24–4
Total Gy	53.8 (4.9)	54.1 (7.4)	62.5–32.4	64–30
Fractions	29.2 (3.1)	28.4 (4.5)	34–18	35–10
Total length (days)	43.6 (8.0)	44.5 (11.8)	31–71	25–110

*SD* standard deviation, *Gy* Gray

### Clinical outcomes

**Fig. 1:**
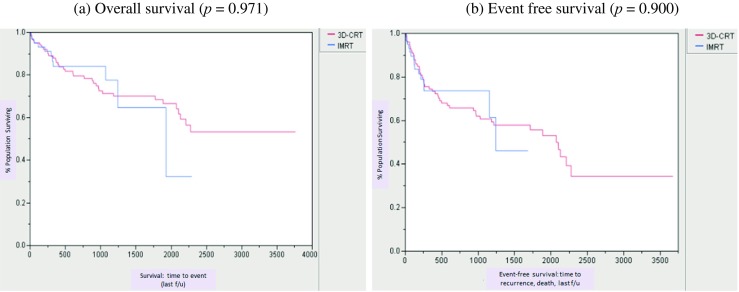
Kaplan-Meier analysis of overall survival and event-free survival by treatment modality

The mean and median follow-up after IMRT and 3D-CRT was 20 months (0.33–76) and 14.7 months, and 47.4 months (1–125) and 46.1 months, respectively. In the IMRT group, rates of CR, PR, and PD were 83.6, 8.2, and 8.2%, respectively, very similar to those among patients treated with 3D-CRT, which were 85.6, 6.7, and 7.7%, respectively (*p* = 0.608, *p* = 0.728, *p* = 0.729). Median overall survival (OS) was not reached for the IMRT group and was 64.2 months for 3D-CRT. There was no statistically significant difference in OS and event-free survival (EFS) (*p* = 0.971, *p* = 0.900) (Fig. 1). Local recurrences were found in eight (13.1%) and seven (6.7%) patients in the IMRT and 3D-CRT groups, respectively (*p* = 0.118). Six (9.8%) and 15 (14.4%) of IMRT and 3D-CRT patients respectively recurred distantly (*p* = 0.373), the majority of which were to liver, lung, perirectal, and inguinal lymph nodes.

### Toxicity

IMRT resulted in less acute high grade (3–4) toxicity, reaching statistical significance for pelvic pain (*p* = 0.033) and fatigue (*p* = 0.030). Decreased rates of high-grade dermatitis (*p* = 0.067), incontinence (*p* = 0.094), and hematologic abnormalities (*p* = 0.239) resulted from IMRT as well. There were no reported instances of grade 3 fatigue or incontinence with IMRT. In both groups, grade 4 toxicity was minimal, three instances (4.9%) in the IMRT group and seven instances (6.8%) in the 3D-CRT group. During the 8-week period following treatment, the 3D-CRT group experienced seven instances of grade 3 toxicity, compared to one instance after IMRT. These were all similar in nature to the late-developing toxicities that occurred from 8 weeks post treatment through 1 year. Hematologic toxicity and dermatitis were the most prevalent high-grade acute toxicities for both IMRT and 3D-CRT (Table 3). Both groups experienced a high degree of acute low-grade (1–2) toxicities, the most prevalent being grade 2 localized dermatitis. IMRT resulted in significantly less low-grade bladder/bowel incontinence compared to 3D-CRT (*p* = 0.035). While not statistically significant, IMRT also resulted in fewer occurrences of grade 2 localized pain, 32.8 vs 45.2% (*p* = 0.115).

**Table 3:** Acute toxicities from chemoradiation treatment

	Value (%)							
	Grade 1		Grade 2		Grade 3		Grade 4	
	IMRT	3D-CRT	IMRT	3D-CRT	IMRT	3D-CRT	IMRT	3D-CRT
Localized pain	25 (41.0)	28 (26.9)	20 (32.8)	47 (45.2)	2 (3.3)	12 (11.5)	0 (0)	1 (1.0)
Diarrhea	19 (31.1)	38 (36.5)	14 (23.0)	26 (25)	4 (6.6)	8 (7.7)	0 (0)	1 (1.0)
Fatigue	20 (32.8)	32 (30.8)	15 (24.6)	23 (22.1)	0 (0)	5 (4.8)	0 (0)	0 (0)
Dermatitis	16 (26.2)	22 (21.2)	37 (60.7)	58 (55.8)	5 (8.2)	19 (18.3)	0 (0)	0 (0)
Hematologic	19 (31.1)	43 (41.3)	14 (23)	24 (23.1)	4 (6.6)	15 (14.4)	3 (4.9)	4 (3.8)
Incontinence	12 (19.7)	30 (28.8)	5 (8.2)	16 (15.4)	0 (0)	2 (1.9)	0 (0)	1 (1.0)
Gastroenteritis^a^	0 (0)	0 (0)	0 (0)	0 (0)	1 (1.0)	1 (1.0)	0 (0)	0 (0)
Colitis	0 (0)	0 (0)	0 (0)	0 (0)	0	2 (1.9)	0 (0)	0 (0)
Proctitis	0 (0)	0 (0)	0 (0)	0 (0)	0	1 (1.0)	0 (0)	0 (0)
Fistula	0 (0)	0 (0)	0 (0)	0 (0)	0	2 (1.9)	0 (0)	0 (0)
Stenosis	0 (0)	0 (0)	0 (0)	0 (0)	0	1 (1.0)	0 (0)	0 (0)

*IMRT* intensity-modulated radiation therapy, *3D-CRT* 3-dimensional conventional radiation therapy

^a^From treatment completion through 8 weeks post treatment

Overall, there were significantly fewer late-developing post-treatment toxicities caused by IMRT (4 occurrences, 6.6% of patients) within 1 year of treatment completion vs 3D-CRT (18 occurrences, 15.4% of patients) (*p* = 0.008). This trend was apparent in every toxicity category assessed except for anal ulcers and stenosis, which was reported in one and two instances, respectively, in each of the two treatment groups. Among 3D-CRT patients, five patients reported proctitis and five patients reported localized pain (Table 6).

Limiting the comparison of toxicity to patients who received MMC chemotherapy resulted in decreased rates of acute and chronic toxicity in the IMRT group comparable to those from the complete group analysis (Tables 4 and 7). IMRT continued to yield lower acute high-grade pain (2 vs 12 occurrences, 4.3 vs 13.5%, *p* = 0.078) and fatigue (0 vs 2 occurrences, 0 vs 2.2%, *p* = 0.034), as well as decreased rates of high-grade dermatitis (5 vs 18 occurrences, 10.9 vs 20.2%, *p* = 0.158), incontinence (0 vs 3 occurrences, 0 vs 3.3%, *p* = 0.111), and hematologic toxicity (6 vs 17 occurrences, 13 vs 19.1%, *p* = 0.366). Grade 4 toxicity remained minimal, with one less occurrence relative to the complete analysis in the 3D-CRT group. IMRT also continued to yield decreased low-grade incontinence (14 vs 42 occurrences, 30.4 vs 47.2%, *p* = 0.059) and grade 2 pain (16 vs 39 occurrences, 34.8 vs 43.8%, *p* = 0.309). Long-term toxicity results were similar to the complete analysis as well. One less instance of stenosis after IMRT and pain after 3D-CRT were recorded, totaling 6.5 and 19.1% of patients (3 vs 17 occurrences), respectively (*p* = 0.078).

**Table 4:** Acute toxicities from chemoradiation treatment—patients who received mitomycin-C (IMRT 75.4%; 3D-CRT 85.6%)

	Value (%)							
	Grade 1		Grade 2		Grade 3		Grade 4	
	IMRT	3D-CRT	IMRT	3D-CRT	IMRT	3D-CRT	IMRT	3D-CRT
Localized pain	19 (41.3)	26 (29.2)	16 (34.8)	39 (43.8)	2 (4.3)	11 (12.4)	0 (0)	1 (1.1)
Diarrhea	15 (32.6)	34 (38.2)	8 (17.4)	23 (25.8)	4 (8.7)	7 (7.9)	0 (0)	0 (1.1)
Fatigue	15 (32.6)	28 (31.5)	10 (21.7)	22 (24.7)	0 (0)	2 (2.2)	0 (0)	0 (0)
Dermatitis	11 (23.9)	14 (15.7)	30 (65.2)	54 (60.7)	5 (10.9)	18 (20.2)	0 (0)	0 (0)
Hematologic	15 (32.6)	36 (40.4)	10 (21.7)	22 (24.7)	3 (6.5)	13 (14.6)	3 (6.5)	4 (4.5)
Incontinence	11 (23.9)	28 (31.5)	3 (6.5)	14 (15.7)	0 (0)	2 (2.2)	0 (0)	1 (1.1)
Gastroenteritis^a^	0 (0)	0 (0)	0 (0)	0 (0)	1 (2.2)	1 (1.1)	0 (0)	0 (0)
Colitis	0 (0)	0 (0)	0 (0)	0 (0)	0	2 (2.2)	0 (0)	0 (0)
Proctitis	0 (0)	0 (0)	0 (0)	0 (0)	0	1 (1.1)	0 (0)	0 (0)
Fistula	0 (0)	0 (0)	0 (0)	0 (0)	0	1 (1.1)	0 (0)	0 (0)
Stenosis	0 (0)	0 (0)	0 (0)	0 (0)	0	1 (1.1)	0 (0)	0 (0)

*IMRT* intensity-modulated radiation therapy, *3D-CRT* 3-dimensional conventional radiation therapy

^a^From treatment completion through 8 weeks post treatment

Of patients who received IMRT, 27.9% had at least one unplanned treatment break, while 41.3% in the 3D-CRT group required a treatment interruption (*p* = 0.89). Patients receiving 3D-CRT missed a mean 4.1 days, compared with 2.9 days for IMRT patients (*p* = 0.229). Of 3D-CRT recipients, 17.3% missed ≥ 10 days, compared to 9.8% of IMRT recipients (*p* = 0.178).

## Discussion

We conducted the largest single-institution study directly comparing outcomes and toxicities of patients treated with 3D-CRT vs IMRT and concurrent 5-FU/MMC for biopsy-proven anal cancer. No statistical significance in OS or EFS emerged between the two groups. IMRT, however, resulted in significantly less acute and late toxicity. While the percentage of patients who required treatment breaks was similar, the IMRT group had shorter breaks (4.1 vs 2.9 days), as well as required fewer extensive (≥ 10 days) breaks compared to the 3D-CRT group.

The relatively brief long-term follow-up time of this study likely limited the statistical significance of OS and EFS as clinical outcomes measures. However, although OS and EFS are often used to assess treatment efficacy, they may not be relevant markers in the context of anal cancer as local recurrences can be salvaged with APR, with a 75% 5-year survival when clean margins are achieved [22]. Survival analyses, therefore, are confounded by the bias introduced by a successful salvage treatment and, as such, are largely measures of death by other causes. It is, therefore, difficult to extrapolate the degree to which reported OS and EFS advantages of IMRT are truly indicative of increased efficacy. Other, more specific end points that have been utilized in large prospective trials include toxicity, locoregional control, complete locoregional response, and progression-free survival [23].

The primary advantage of IMRT over 3D-CRT in our series was reduced acute and late toxicity, in particular high-grade toxicity (Table 3). These outcomes were notwithstanding the higher mean pelvic dose delivered with IMRT (48.3 Gy) compared to 3D-CRT (44 Gy). While some reports, including from the RTOG, recommended a pelvic dose of 30.6 Gy for conformal radiotherapy due to concerns for toxicity [24–26], doses of 40–45 Gy are commonly prescribed. The prospective trial RTOG 05–29 revealed a significant reduction in grade ≥ 3 dermatologic and gastrointestinal and grade ≥ 2 hematologic toxicities using IMRT to deliver 42–45 Gy to the pelvis before boost [20].

The most prevalent high-grade acute toxicity in both groups was hematologic (11.5 and 18.2% for IMRT and 3D-CRT, respectively), primarily leukopenia. This may have been due in part to the known immunosuppressant effects of MMC, which 84.8% of patients received, as well as inclusion of the broad pelvic bones in the radiation field [27]. IMRT, which limits the dose delivered to the greatest regions of pelvic bone marrow, had a lower rate compared with 3D-CRT. Of note, controlling for the presence of MMC resulted in nearly identical rates of toxicity compared to the whole group analysis in which fewer IMRT patients received MMC (75.4%) than did 3D-CRT patients (85.6%) (Table 5). Other studies found high rates of grade 3–4 hematologic toxicity, for example, 61% in RTOG 98-11 using conventional techniques [28], and 31% reported by Pepek et al. with IMRT (47 patients, no direct 3D-CRT comparison) [16]. Milano et al. found no benefit to IMRT (17 patients) relating to hematologic toxicity compared to 3D-CRT (7 patients) (rates not reported) [11].

**Table 5:** Change in percent (%) acute toxicity: Total group ➔ patients who received mitomycin-C (IMRT 75.4%; 3D-CRT 85.6%)

	Grade 1		Grade 2		Grade 3		Grade 4	
	IMRT	3D-CRT	IMRT	3D-CRT	IMRT	3D-CRT	IMRT	3D-CRT
Localized pain	41 ➔ 41.3	26.9 ➔ 29.2	32.8 ➔ 34.8	45.2 ➔ 43.8	3.3 ➔ 4.3	11.5 ➔ 12.4	0	1 ➔ 1.1
Diarrhea	31.1 ➔ 32.6	36.5 ➔ 38.2	23 ➔ 17.4	25 ➔ 25.8	6.6 ➔ 8.7	7.7 ➔ 7.9	0	1 ➔ 1.1
Fatigue	32.8 ➔ 32.6	30.8 ➔ 31.5	24.6 ➔ 21.7	22.1 ➔ 24.7	0	4.8 ➔ 2.2	0	0
Dermatitis	26.6 ➔ 23.9	21.2 ➔ 15.7	60.7 ➔ 65.2	55.8 ➔ 60.7	8.2 ➔ 10.9	18.3 ➔ 20.2	0	0
Hematologic	31.1 ➔ 32.6	41.3 ➔ 40.4	23 ➔ 21.7	23.1 ➔ 24.7	6.6 ➔ 6.5	14.4 ➔ 14.6	4.9 ➔ 6.5	3.8 ➔ 4.5
Incontinence	19.7 ➔ 23.9	28.8 ➔ 31.5	8.2 ➔ 6.5	15.4 ➔ 15.7	0	1.9 ➔ 2.2	0	1 ➔ 1.1
Gastroenteritis^a^	0	0	0	0	1 ➔ 2.2	1 ➔ 1.1	0	0
Colitis	0	0	0	0	0	1.9 ➔ 2.2	0	0
Proctitis	0	0	0	0	0	1 ➔ 1.1	0	0
Fistula	0	0	0	0	0	1.9 ➔ 1.1	0	0
Stenosis	0	0	0	0	0	1 ➔ 1.1	0	0

*IMRT* intensity-modulated radiation therapy, *3D-CRT* 3-dimensional conventional radiation therapy

^a^From treatment completion through 8 weeks post treatment

Toxicity rates from both IMRT and 3D-CRT in our study were also lower than those presented in previous studies. In the 2008 report from RTOG 98-11, 48% of patients receiving 3D-CRT and 5-FU/MMC had grade 3–4 dermatologic toxicity based on the CTCAE v2.0 [28], compared with 18.3% grade 3 toxicity in our series. Kachnic et al. reported a 20% rate of grade ≥ 3 dermatologic toxicity, as well as 22% of patients experiencing grade ≥ 3 gastrointestinal toxicity for IMRT [29] (43 patients, no direct 3D-CRT comparison), compared with 8.2 and 6.6% in our study. Pepek et al. reported rates of 9% grade 3 and no grade 4 diarrhea, comparable to our findings [16].

IMRT reduced toxicity up to 1 year following the end of chemoradiation (Table 6). While few studies have investigated post-treatment toxicity, even fewer have reported on specific categories of long-term sequelae [13]. In our series, notable differences emerged for proctitis (0 vs 5) and pain (0 vs 5), with IMRT demonstrating fewer chronic toxicities in six of eight categories analyzed. Such chronic sequelae are debilitating and substantially contribute to the cost of disease management. While analyzing influences on treatment breaks and toxicity from radiation for gastrointestinal malignancies, Hill et al. reported a strong correlation between toxicity and unplanned hospital admissions [30], which inevitably entails added resources and services. In contrast, Hodges et al., based on a novel Markov decision model designed to assess both cost and quality of life discrepancies between patients with anal cancer treated with IMRT vs 3D-CRT, concluded a cost-ineffectiveness of IMRT despite its lower overall risk of acute toxicity [31].

**Table 6:** Late toxicities within 1 year of treatment

	Number of occurrences	
	IMRT	3D-CRT
Diarrhea	1	2
Stenosis	2	2
Enterocolitis	0	1
Proctitis	0	5
Anal ulcer	1	1
Fistula	0	1
Wound dehiscence	0	1
Pain	0	5
Total occurrences	4	18
% of patients	6.6	15.4

*IMRT* intensity-modulated radiation therapy, *3D-CRT* 3-dimensional conventional radiation therapy

In our analysis, there was a trend towards fewer and shorter treatment breaks for IMRT patients compared with 3D-CRT. Recent studies have suggested that treatment breaks are associated with poorer clinical outcomes: RTOG 92-08, which included a pre-specified 2-week treatment break, resulted in decreased overall, disease-free, and colostomy-free survival compared to trials with no treatment breaks [32]. Similarly, Roohipour et al. and investigators of the ACT II trial both reported that treatment breaks contributed to worse OS, relapse-free survival, and local control [33, 34]. Other series, however, failed to show any correlation between treatment duration, breaks and outcome [12, 35]. In our series, IMRT patients experienced significantly fewer instances of high-grade toxicities, likely resulting in their tendency to need shorter interruptions. Rates of low-grade toxicity were only slightly reduced with IMRT, perhaps resulting in the comparable need for short treatment breaks (Table 7).

**Table 7:** Late toxicities within 1 year of treatment—patients who received mitomycin-C (*IMRT* 75.4%; 3D-CRT 85.6%)

	Number of occurrences	
	IMRT	3D-CRT
Diarrhea	1	2
Stenosis	1	2
Enterocolitis	0	1
Proctitis	0	5
Anal ulcer	1	1
Fistula	0	1
Wound dehiscence	0	1
Pain	0	4
Total occurrences	3	17
% of patients	6.5	19.1

*IMRT* intensity-modulated radiation therapy, 3D-CRT 3-dimensional conventional radiation therapy

Studies that found a positive correlation between treatment breaks and worse outcomes have concluded that interruptions can be used as a quantitative surrogate for toxicity, based on the assumption that unplanned breaks are an opportunity to recover from an acute radiation-induced toxicity. Differences in physicians’ willingness to continue treatment despite toxicities, unique institutional protocols, and vigilance in toxicity prevention, however, all contribute variability. Additionally, some institutions elect to treat over weekends, or may lack the ability to continue treatment in spite of a hospital admission or other need. These considerations weaken the ability to directly equate toxicity with treatment interruption both between and within institutions.

Possible limitations of our analysis include heterogeneity in the exact chemotherapy and radiation given, as well as inter-rater variability of physician-reported toxicity. Both potential biases, however, should be equally present in both groups. Furthermore, IMRT was gradually adopted as the primary treatment modality for anal cancer at our institution (2006–2012) while 3D-CRT was being utilized (2003–2012), which should have limited differences in the learning curve or level of caregiver training between treatment types. Another limitation of our study is the relatively short follow-up time of IMRT (median 14.7 months) limiting the reporting of late toxicity to 1 year post treatment. In order to perform the most robust toxicity analysis possible with the greatest number of patients, we were limited to long-term follow-up of 1 year. In the context of a comparison of chronic toxicity, however, this is a relatively short time. While a clear trend of decreased post-treatment toxicity was established, a longer follow-up comparison would likely reveal more definitive results. Additional prospective studies with longer follow-up time following IMRT would further elucidate its benefits.

## Conclusions

In conclusion, IMRT for anal cancer treatment results in reduced acute and late toxicity compared to 3D-CRT. IMRT also allows for safe dose escalation with survival and recurrence outcomes similar to 3D-CRT. Patients who received IMRT required less extensive treatment breaks. A decreased rate of grade 3–4 toxicity may help reduce the need for costly therapies and services to address side effects.
